# Dysregulation of intercellular signaling by MOF deletion leads to liver injury

**DOI:** 10.1074/jbc.RA120.016079

**Published:** 2021-01-07

**Authors:** Hongwei Lei, Aaron D. denDekker, Guobing Li, Zhiguo Zhang, Liang Sha, Matthew A. Schaller, Steven L. Kunkel, Liangyou Rui, Kaixiong Tao, Yali Dou

**Affiliations:** 1 Department of Gastrointestinal Surgery, Union Hospital, Tongji Medical College, Huazhong University of Science and Technology, Wuhan, China; 2 Department of Medicine, University of Southern California, Los Angeles, California, USA; 3 Department of Surgery, University of Michigan, Ann Arbor, Michigan, USA; 4 Department of Molecular & Integrative Physiology, University of Michigan Medical School, Ann Arbor, Michigan, USA; 5 Division of Pulmonary, Critical Care & Sleep Medicine, University of Florida, Gainesville, Florida, USA

**Keywords:** histone acetyltransferase, MOF, epigenetics, liver injury, steatohepatitis, mitochondria dysregulation, fibrosis, epigenetic regulation, ALKP, alkaline phosphatase, ALT, alanine aminotransferase, AST, aspartate aminotransferase, BMDM, bone marrow–derived macrophage, CFD, choline- and folate-deficient, FDR, false discovery rate, GO, gene ontology, H&E, hematoxylin and eosin, HAT, histone acetyltransferase, HCC, hepatocelluar carcinoma, IHC, immunohistochemistry, iNOS, inducible nitric oxide synthase, LDH, lactate dehydrogenase, MOF, males absent on the first, NAFLD, nonalcoholic fatty liver disease, NASH, nonalcoholic steatohepatitis, NGS, normal goat serum, RT, room temperature, SNP, sodium nitroprusside, TBG, thyroxine-binding globulin, TCA, tricarboxylic acid, TLR, toll-like receptor, TNF, tumor necrosis factor

## Abstract

Epigenetic mechanisms that alter heritable gene expression and chromatin structure play an essential role in many biological processes, including liver function. Human MOF (males absent on the first) is a histone acetyltransferase that is globally downregulated in human steatohepatitis. However, the function of MOF in the liver remains unclear. Here, we report that MOF plays an essential role in adult liver. Genetic deletion of *Mof* by Mx1-Cre in the liver leads to acute liver injury, with increase of lipid deposition and fibrosis akin to human steatohepatitis. Surprisingly, hepatocyte-specific *Mof* deletion had no overt liver abnormality. Using the *in vitro* coculturing experiment, we show that *Mof* deletion-induced liver injury requires coordinated changes and reciprocal signaling between hepatocytes and *Kupffer* cells, which enables feedforward regulation to augment inflammation and apoptotic responses. At the molecular level, *Mof* deletion induced characteristic changes in metabolic gene programs, which bore noticeable similarity to the molecular signature of human steatohepatitis. Simultaneous deletion of *Mof* in both hepatocytes and macrophages results in enhanced expression of inflammatory genes and NO signaling *in vitro*. These changes, in turn, lead to apoptosis of hepatocytes and lipotoxicity. Our work highlights the importance of histone acetyltransferase MOF in maintaining metabolic liver homeostasis and sheds light on the epigenetic dysregulation in liver pathogenesis.

Emerging evidence shows that epigenetic mechanism converts alterations in nutrient and metabolism into heritable patterns of gene expression and has profound implications in human physiology and diseases (1, 2). Extensive interplays between epigenetic regulation and cell metabolism are reported to influence various cellular processes (3, 4). For instance, the tricarboxylic acid (TCA) cycle generates by-products such as acetyl-CoA and S-adenosyl-methionine that are substrates of histone-modifying enzymes (3, 5). Histone modifications, in turn, regulate expression of important metabolic genes that are critical for the catabolic and anabolic processes to support cell survival and growth. Histone modifications also directly modulate cell signaling to ensure the balance of nutrient availability and cellular capacity to use them effectively. Surprisingly, despite the prominent role of the liver in all metabolic processes in the body, there is a paucity of studies investigating deregulation of histone modifications and histone-modifying enzymes in the liver and their roles in common liver diseases. Among histone modifications, it is reported that global change of histone acetylation is associated with the progression of cirrhosis (6). Furthermore, altered expression or activity of the histone deacetylases (*e.g.*, HDAC3) and sirtuins (*e.g.*, SIRT1) is implicated in aberrant hepatic metabolism and progression of nonacoholic fatty liver disease (NAFLD) (7, 8). These studies suggest that histone acetylation may play an important role in the liver. However, the physiological and pathological functions of histone acetyltransferases (HATs) have not been directly examined in the liver.

Among the histone acetyltransferases, males absent on the first (*Mof*, also called KAT8 or MYST1) is highly conserved and plays a nonredundant function in depositing lysine (K) 16 acetylation on histone H4 (H4K16ac) (9, 10). H4K16ac is a prerequisite for additional H4 acetylation and higher-order chromatin structure and is associated with transcription activation (11, 12). We and others have shown that *Mof* plays important function in embryonic stem cell (ESC) self-renewal (13, 14), DNA damage repair (15, 16), senescence (17), and autophagy (18). *Mof* also regulates fatty acid oxidation and mitochondria respiration. *Mof* deletion in ground-state ESCs leads to pluripotent quiescence by blocking fatty acid oxidation pathways (19). *Mof* depletion in cardiomyocytes increases reactive oxidative species (ROS) as a result of mitochondria dysregulation (20). *In vivo* studies show that deletion of *Mof* in the *Mof* ^*f/f*^; ER-Cre mouse model results in lethality in adult mice with postmortem liver abnormality (21). Significant reduction of MOF protein is found in a choline- and folate-deficient (CFD) mouse model of nonalcoholic steatohepatitis (NASH) (22). Lower level of H4K16ac is associated with poor overall disease-free survival of hepatocelluar carcinoma (HCC) patients (23, 24). Despite these studies, the causal function of *Mof* in the liver has not been directly studied.

To examine the function of *Mof* in the liver, the main organ for metabolic processes in the body, we genetically deleted *Mof* in the liver. We find that simultaneous deletion of *Mof*, by Mx1-Cre, in multiple cell compartments in the liver leads to acute liver injury with increase of fat deposition, liver fibrosis, and cell death. Interestingly, the pleiotropic defects are not observed in mice with specific *Mof* deletion in hepatocytes. Mechanistic studies show that *Mof* deletion in both hepatocytes and *Kupffer* cells is necessary for liver pathogenesis and leads to feedback augmentation of inflammation and apoptosis signaling in the liver microenvironment. Consistent with importance of *Mof* in the liver, we find that *MOF* is frequently downregulated in human NASH patients and that *Mof*-dependent gene program is often deregulated in this deadly liver disease (25, 26). Taken together, our results show that deregulation of *Mof* is likely a novel contributor to metabolic liver diseases.

## Results

### Establishment of the Mx1-Cre; *Mof*^*f/f*^ mouse model

**Figure 1:**
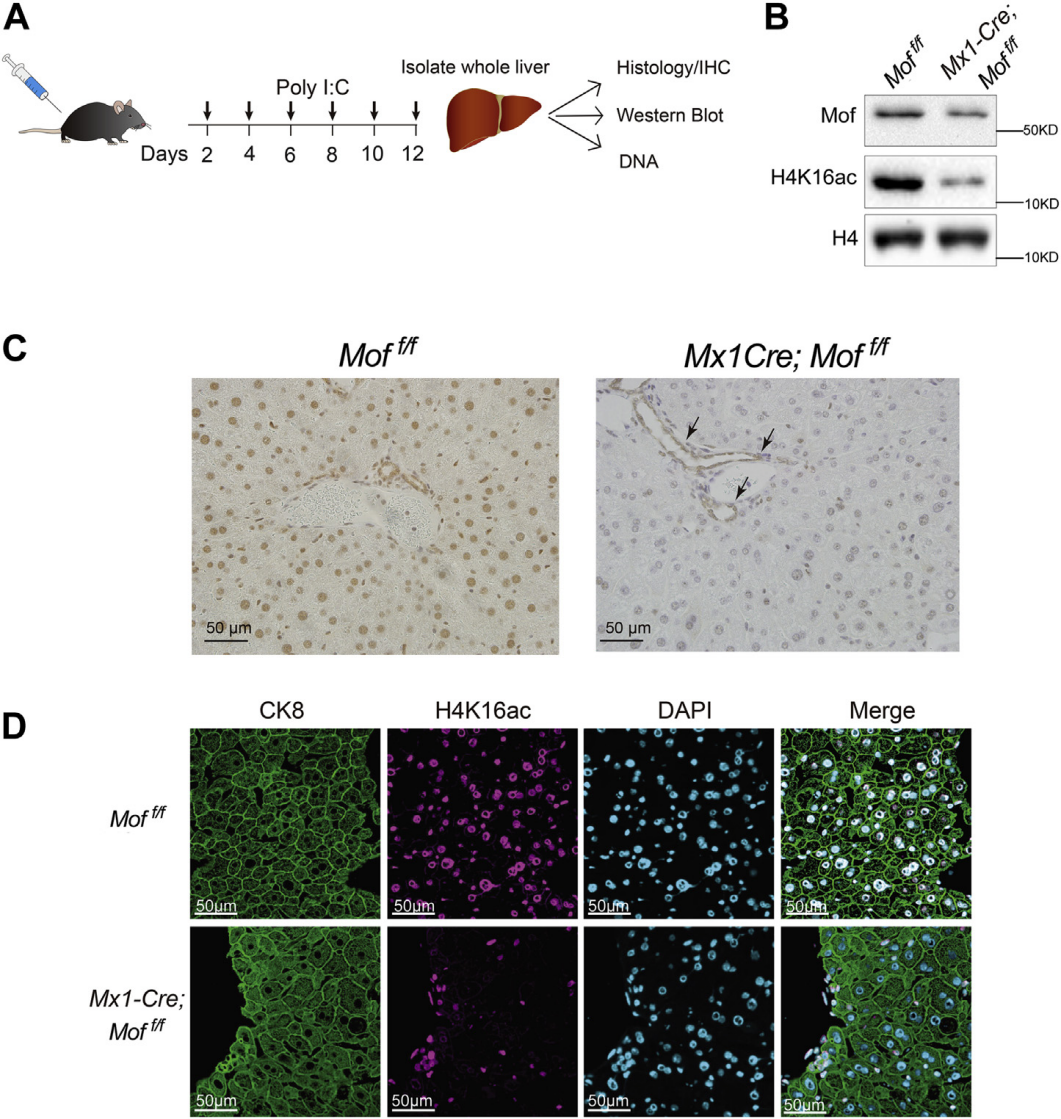
**Inducible deletion of *Mof* by Mx1-Cre.***A*, an outline of the experimental strategy. *B*, western blot for MOF and H4K16ac in hepatocytes isolated from polyI:C-treated *Mof*^*f/f*^ (no Cre recombinase) and Mx1-Cre; *Mof*^*f/f*^ livers. *C*, representative immunohistochemistry for MOF protein in livers of the polyI:C treated *Mof*^*f/f*^ (no Cre recombinase) and *Mx1-Cre*; *Mof*^*f/f*^ mice, as indicated on *top*. *Arrows* denote cholangiocytes and endothelial cells that escaped *Mof* deletion. Scale bar, 50 μm. *D*, representative immunofluorescence images for CK8 and H4K16ac staining in polyI:C-treated adult livers from *Mof*^*f/f*^ and Mx1-Cre; *Mof*^*f/f*^ mice following treatment.

To study the function of MOF, we generated the Mx1-Cre; *Mof* ^*f/f*^ mouse model (Fig. S1*A*), which deletes *Mof* in multiple cellular compartments including hepatocytes and *Kupffer* cells (27, 28). In this model, *Mof* was efficiently deleted at day 12 post poly-inosinic:poly-cytadilic acid (polyI:C) treatment (Fig. 1*A* and Fig. S1*A*). Consistent with *Mof* deletion, both MOF protein and cognate histone H4 K16 acetylation (H4K16ac) were greatly reduced in the *Mof* ^*−/−*^ liver (Fig. 1*B* and Fig. S1*B*). No *Mof* deletion was detected in the livers of the control polyI:C-treated *Mof* ^*f/f*^ or Mx1-Cre; *Mof* ^*+/+*^ mice (Fig. S1*A*). To further confirm *Mof* deletion, we performed immunohistochemistry (IHC) for MOF in the livers of *Mof* ^*f/f*^ and Mx1-Cre; *Mof* ^*f/f*^ mice after polyI:C treatment. As shown in Figure 1*C*, MOF was largely absent in the nuclei of liver cells from the Mx1-Cre; *Mof* ^*f/f*^ mice. MOF was retained in a small subset of liver cells including endothelial cells and cholangiocytes surrounding veins and bile ducts as expected (Fig. 1*C*). Immunofluorescence for H4K16ac further confirmed loss of MOF in majority of liver cells (Fig. 1*D*). Since Mx1-Cre is also expressed in the hematopoietic system (27, 29), we measured levels of lineage-committed hematopoietic cells in the peripheral blood and bone marrow upon *Mof* deletion. We did not observe significant changes in the levels of T cells (CD3^+^), B cells (B220^+^), or myeloid cells (Gr1^+^, CD11b^+^) in the peripheral blood (Fig. S1*C*). Similarly, no significant change in the hematopoietic progenitors was detected in the bone marrow for up to day 60 post polyI:C treatment (Fig. S1*C* and data not shown).

### *Mof* deletion leads to acute liver injury

**Figure 2:**
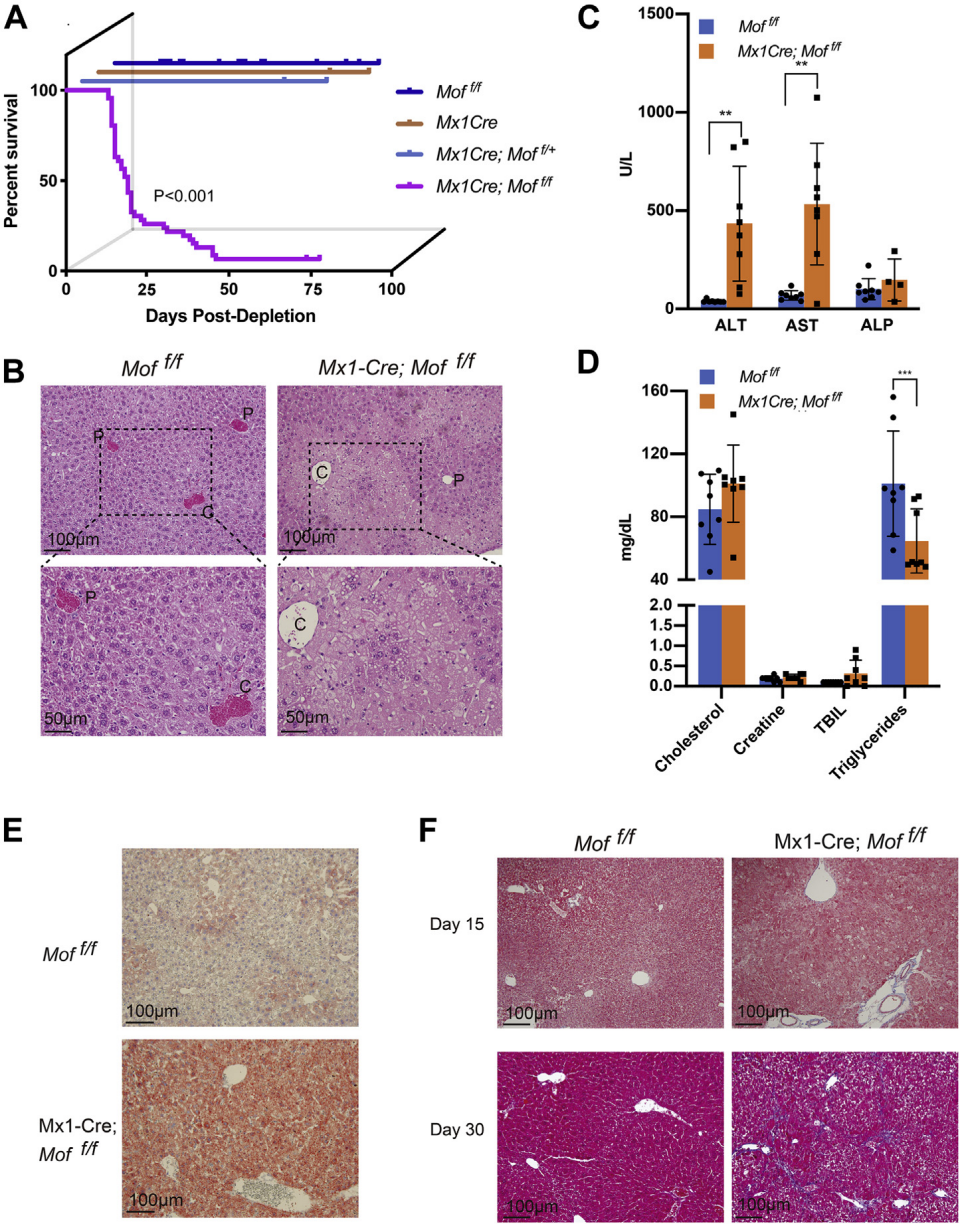
***Mof* deletion in adult livers led to terminal liver failure.***A*, Kaplan–Meier survival curve post polyI:C treatment. Mouse genotypes were indicated on the right. Log-rank test was performed and *p* < 0.001 for poly I:C-treated Mx1-Cre; *Mof*^*f/f*^ mice. A total of 44 male and 43 female mice were used for each group. *B*, representative H & E staining of polyI:C-treated adult livers from *Mof*^*f/f*^ and Mx1-Cre; *Mof*^*f/f*^ mice. Pale regions in Mx1-Cre; *Mof*^*f/f*^ tissue are indicative of cell death. C, central vein; P, portal vein. Scale bars, 50 μm (bottom) and 100 μm (*top*), respectively. *C*–*D*, scatter plot of basic liver chemistry panels as indicated on the *bottom*. The samples were analyzed at day 26 post polyI:C treatment. Two-way ANOVA was used for statistical analysis, ∗∗*p* < 0.002. Eight mice per genotype, four males and four females, were used in the analyses. *E*, oil Red O staining for lipid droplets in the polyI:C-treated *Mof*^*f/f*^ and Mx1-Cre; *Mof*^*f/f*^ livers. Scale bars, 100 μm. The staining was performed at day 26 post poly I:C treatment. *F*, Masson’s trichrome staining of *Mof*^*f/f*^ and Mx1-Cre; *Mof*^*f/f*^ livers at day 15 and 30 post polyI:C treatment. Blue staining is indicative of collagen deposition. Scale bars, 100 μm.

Upon polyI:C treatment, approximately 70% of Mx1-Cre; *Mof* ^*f/f*^ mice exhibited labored breathing and slowed gait in 3 weeks and had to be euthanized. The remaining Mx1-Cre; *Mof* ^*f/f*^ mice eventually succumbed within 60 days (Fig. 2*A*). In contrast, the polyI:C-treated control Mx1-Cre; *Mof* ^*f/+*^, *Mof* ^*+/+*^ and *Mof* ^*f/f*^ (no Cre) mice were normal at the study end point (80 days) (Fig. 2*A*). Livers from moribund Mx1-Cre; *Mof* ^*f/f*^ mice were significantly enlarged as compared with those of the control mice (Fig. S2*A*). In some cases (2 out of 20), *Mof* ^*−/−*^ livers were visibly whitened due to significantly increased lipid deposition (Fig. S2*B*). Histology of the *Mof* ^*−/−*^ livers showed massive hepatic cell death radiating from the central vein (Fig. 2*B*), suggestive of terminal liver failure. Blood serum levels of liver enzymes, including alanine aminotransferase (ALT), aspartate aminotransferase (AST), lactate dehydrogenase (LDH), bilirubin (TBIL), and alkaline phosphatase (ALKP), were significantly elevated in *Mof* ^*−/−*^ mice as compared with the polyI:C-treated control *Mof* ^*f/f*^ mice (Fig. 2, *C*–*D*). Since elevation of blood AST and ALT levels is often associated with hepatic steatosis and hepatitis (30), we examined the *Mof* ^*−/−*^ and *Mof* ^*f/f*^ livers for steatohepatitis-like features such as lipid deposition and fibrosis. Strikingly, majority of hepatocytes from *Mof* ^*−/−*^ mice showed enhanced accumulation of lipid microdroplets (Fig. 2*E*). Increase of *Cyp2e1* expression, indicative of lipotoxicity, was also detected in *Mof* ^*−/−*^ livers (Fig. S2*C*). Furthermore, there was significant fibrosis in most *Mof* ^*−/−*^ livers isolated from moribund mice that survived past day 30 of polyI:C treatment (Fig. 2*F*, bottom). Taken together, *Mof* deletion by Mx1-Cre leads to severe liver injury, dysregulation of fatty acid metabolism, and increased liver fibrosis, which are characteristics of steatohepatitis (31).

### MOF regulates gene pathways that are dysregulated in human liver disease.

**Figure 3:**
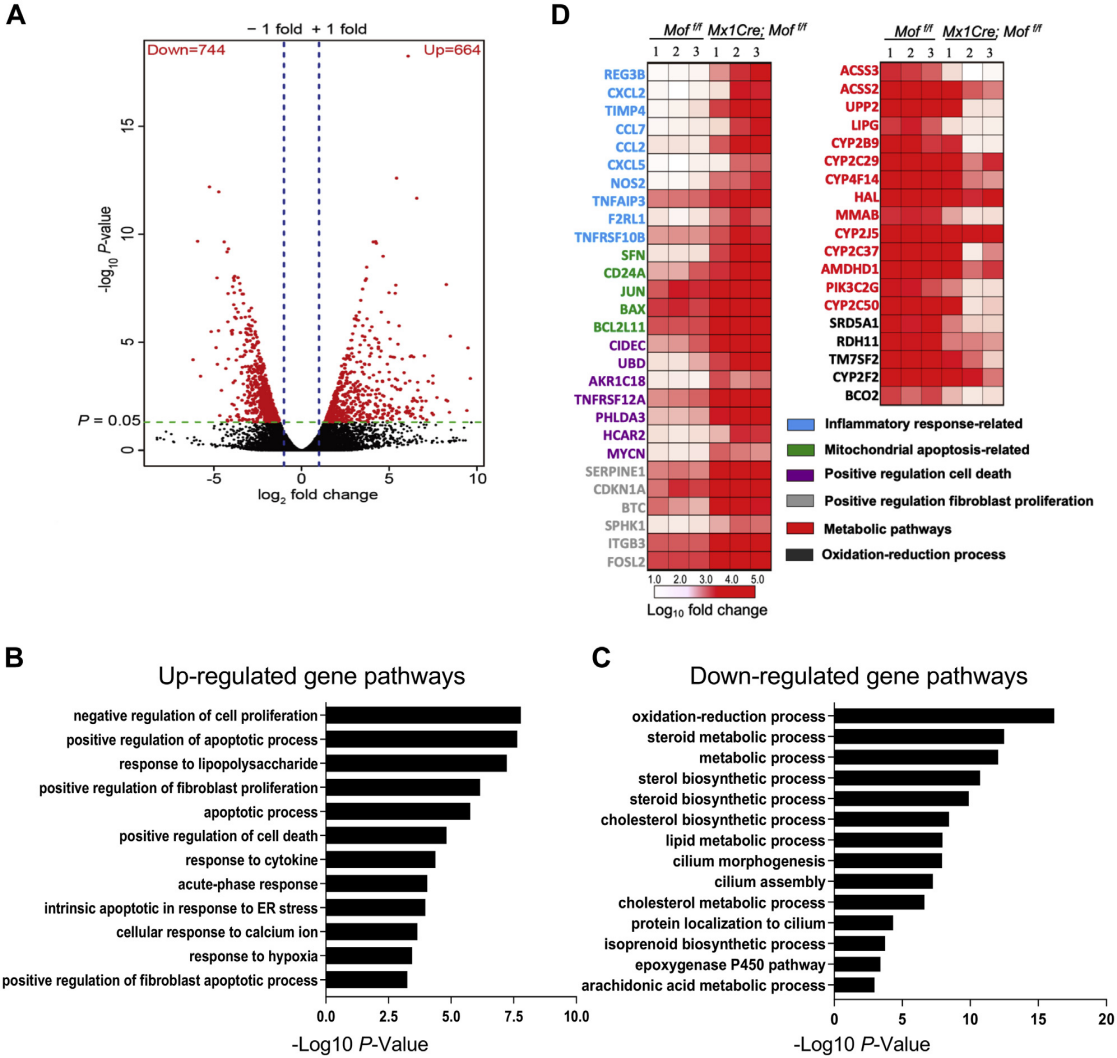
***Mof* regulates important gene pathways for proper liver functions.***A*, volcano plot of genes differentially expressed in *Mof* null livers as compared with the control *Mof^f/f^* liver. *Red dots* depict genes with log2 fold change of ≥ ±1 and *p* value ≤0.05. (N = 3/group). *B*, KEGG analysis of upregulated gene pathways. *C*, KEGG analysis of downregulated gene pathways. *D*, heatmap of expression of representative *Mof* target genes after *Mof* deletion. Heatmap key on bottom indicates normalized log10 fold change. Gene pathway annotation is indicated by color coding.

To examine the function of *Mof* deletion at the molecular level, we performed RNA-seq analyses on primary liver tissues isolated from the *Mof* ^*−/−*^ and *Mof* ^*f/f*^ mice. There was a total of 1408 differentially regulated genes (fold change >2, *p* ≤ 0.05), of which 664 genes were upregulated and 744 genes were downregulated upon *Mof* deletion (Fig. 3*A*). Gene pathway analyses for the upregulated genes showed that they were enriched for pathways such as negative regulation of cell proliferation, apoptotic process, response to lipopolysaccharide, cytokine response, and fibroblast proliferation (Fig. 3*B*), consistent with steatohepatitis-like phenotypes *in vivo* (Fig. 2). Interestingly, the enriched gene pathways for the downregulated genes almost exclusively involve metabolic processes, such as oxidation reduction and the sterol and cholesterol biosynthetic pathways (Fig. 3*C*) (Table S1). GSEA analysis further confirmed enrichment of apoptosis and inflammatory response pathways in the upregulated genes (Fig. S3*A*), as well as enrichment of fatty acid metabolism and other mitochondria processes in the downregulated genes (Fig. S3*B*). Heatmap of representative *Mof* targets in these pathways was shown in Figure 3*D* and expression of selected genes was confirmed by real-time PCR (Fig. S3*C*).

**Figure 4:**
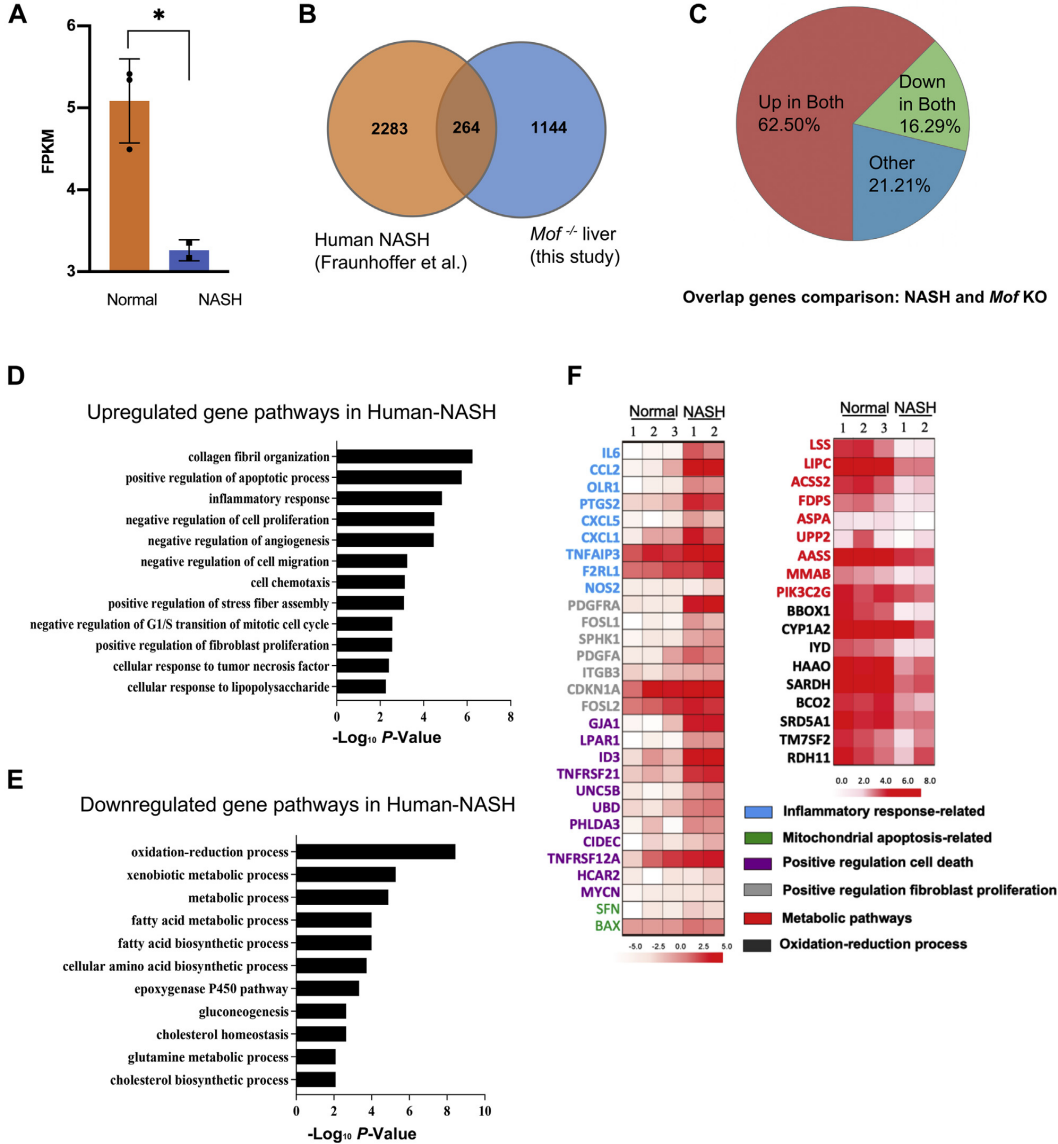
***MOF* and MOF-dependent gene network are dysregulated in human NASH patients.***A*, FPKM expression values of *MOF* in human NASH patients and healthy controls (∗*p* < 0.05, student *t*-test) (GSE134422) (45). *B*, Venn diagram of differentially expressed genes (fold change > 2) in M*of* null mouse livers and human NASH samples. *C*, pie chart for genes dysregulated in both mouse and human datasets. *D*, upregulated biological processes in the human NASH dataset. X-axis shows negative log_10_*p* value. *E*, downregulated biological processes in human NASH dataset. X-axis shows negative log_10_*p* value. *F*, heatmap of expression of representative *Mof* targets in human NASH and healthy control samples as indicated on top. Heatmap key on bottom indicates normalized log_10_ fold change.

Given the steatohepatitis-like features in *Mof* null mice (*e.g.*, increase of fat deposition, fibrosis, elevated serum AST and ALT), we compared gene pathways dysregulated by *Mof* deletion with those in human NASH patients. Interestingly, RNA-seq analyses of primary liver samples from human NASH patients and healthy controls (GSE134422) showed that *MOF* was significantly downregulated in human NASH patients (Fig. 4*A*). Furthermore, there were over 2283 genes with altered expression in human NASH patients. Among them, a significant subset of genes (∼12%) were dysregulated in both human NASH and the *Mof* deletion mouse model (Fig. 4, *B*–*C*). Majority (62.50%) of the commonly deregulated genes were involved in inflammatory response and apoptotic pathways in KEGG pathway analysis (Fig. 4*D*). Pathways such as oxidation–reduction and lipoprotein metabolic pathways were among the commonly downregulated pathways in both human NASH patients and *Mof* null mice (Fig. 4*E*, Table S2). Expression of representative inflammatory signaling factors such as *NOS2*, *CXCL5*, and *CCL2* and metabolic genes such as *ACSS2*, *MMAB*, and *PIK3C2G* in *Mof* null liver and human NASH patients were shown in Figure 4*F*. Similar conclusions could also be drawn from analyzing an independent RNA-seq data set from 16 human NASH patients (Fig. S4, *B*–*D* and Table S3) (31). These results suggest that *Mof* deletion-mediated transcriptome changes carry some molecular characteristics of human NASH.

### Hepatocyte-specific *Mof* deletion had no apparent liver defects

**Figure 5:**
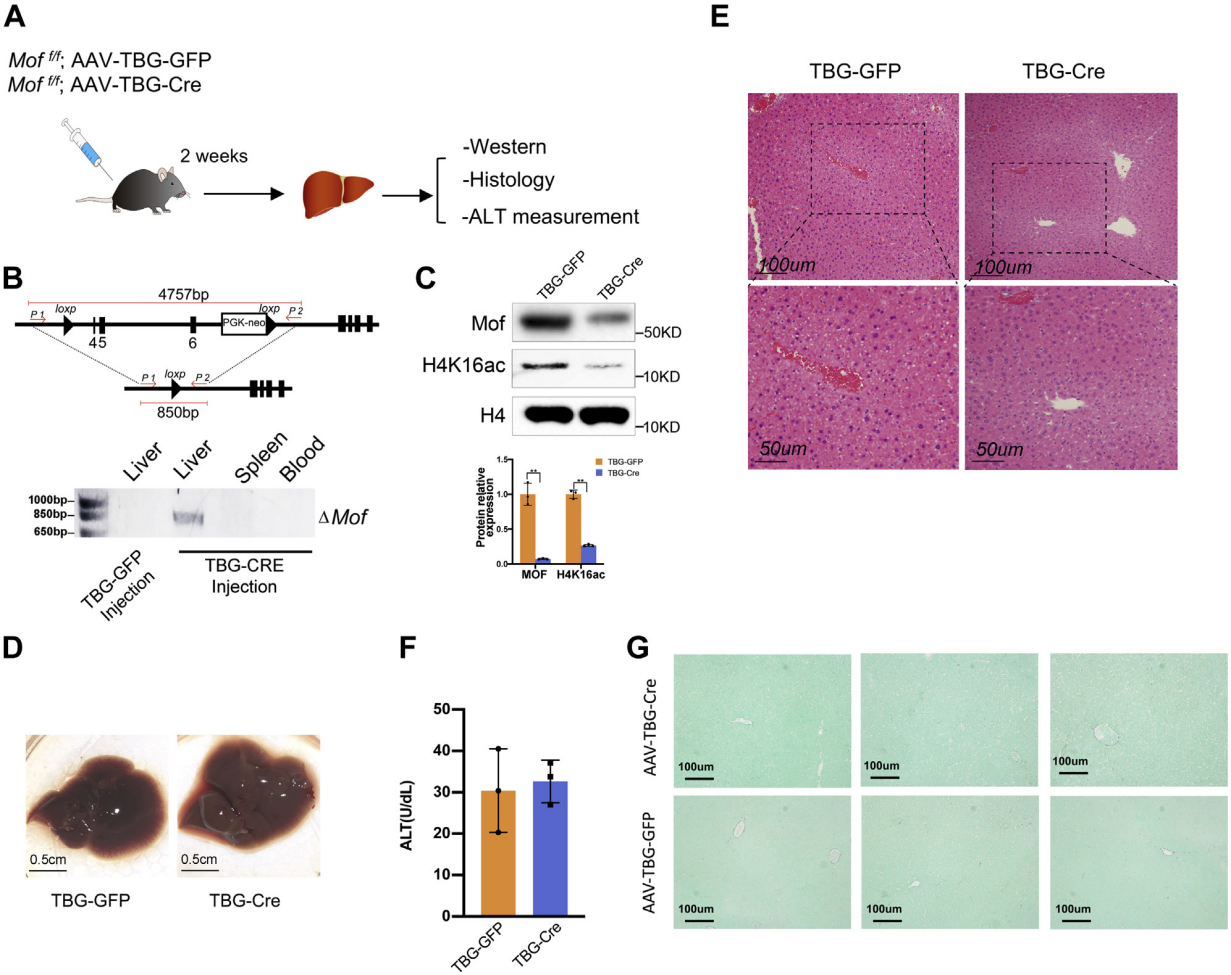
**Hepatocyte-specific *Mof* deletion had no overt phenotype.***A*, schematics for analyzing *Mof* deletion in hepatocyte. *B*, *Top*, genotyping strategy for *Mof*. *Bottom*, the DNA gel for the PCR products using the liver, spleen, and peripheral blood tissues as indicated on *top*. *Mof* deletion was only detected by PCR in livers isolated from AAV-TBG-Cre injected mice. *Mof* deletion was not detected in livers of the control AAV-TBG-GFP injected *Mof*^*f/f*^ mice or in other organs of the AAV-TBG-Cre-injected *Mof*^*f/f*^ mice. *C*, western blot for MOF and H4K16ac in livers isolated from the AAV-TBG-Cre and AAV-TBG-GFP mice as indicated on the left. Western blot for histone H4 was used as the loading control. *D*, representative image for livers isolated from mice at day 26 post either AAV-TBG-Cre or AAV-TBG-GFP injection, scale bar 0.5 cm. *E*, representative H&E staining for livers isolated at day 26 from AAV-TBG-Cre; *Mof*^*f/f*^ and AAV-TBG-GFP; *Mof*^*f/f*^ mice. Scale bar, 50 μm (*bottom*) or 100 μm (*top*) as indicated. *F*, level of serum ALT at day 30 post AAV-TBG-GFP or AAV-TBG-Cre injection. Mean and standard deviation (error bars) from five independent experiments were presented. No statistic difference was detected. *G*, representative Sirius Red (Sigma, 365548) and Fast Green (Sigma, F7252) staining for the liver sections from three different mice following either AAV-TBG-GFP or AAV-TBG-Cre injection, scale bar 100 μm.

Since Mx1-Cre is expressed in both hepatocytes and *Kupffer* cells in the liver (28), we decided to examine whether liver failure upon *Mof* deletion is intrinsic to hepatocytes. To this end, we specifically deleted *Mof* in hepatocytes of the *Mof* ^*f/f*^ mice by tail vein injection of adeno-associated virus (AAV) expressing Cre recombinase under the control of the promoter of hepatocyte-specific thyroxine-binding globulin gene (TBG) (AAV-TBG-Cre) (Fig. 5*A*). In parallel, we injected AAV expressing TBG-driven green fluorescent protein (AAV-TBG-GFP) as the negative control (Fig. 5*A*). Injection of AAV-TBG-Cre, but not AAV-TBG-GFP, specifically deleted *Mof* in the liver, but not in hematopoietic organs such as the spleen and peripheral blood, as indicated by the genotyping result for *Mof* excision (Fig. 5*B*) (32, 33). Western blot analysis further confirmed significant reduction of both MOF protein and H4K16ac in the livers after AAV-TBG-Cre injection (Fig. 5*C*). The remanent signals for MOF and H4K16ac were likely from nonhepatocytes (*e.g.*, Kupffer cells) in the liver, which maintained normal *Mof* expression. Surprisingly, unlike the Mx1-Cre mouse model, all mice were viable at least 3 months after AAV-TBG-Cre injection. Livers from mice injected with AAV-TBG-CRE or AAV-TBG-GFP showed no obvious difference. H&E staining showed normal liver architecture in *Mof* ^*−/−*^ mice (Fig. 5*E*). Consistently, serum level of ALT was normal at day 30 post deletion (Fig. 5*F*). No fibrosis was detected in the liver of *Mof* ^*−/−*^ mice (Fig. 5*G*). These results suggest that hepatocyte-specific *Mof* deletion does not have detrimental effects in the liver.

### Simultaneous deletion of *Mof* in hepatocytes and bone marrow–derived macrophages (BMDMs) increases apoptosis of hepatocytes *in vitro*

**Figure 6:**
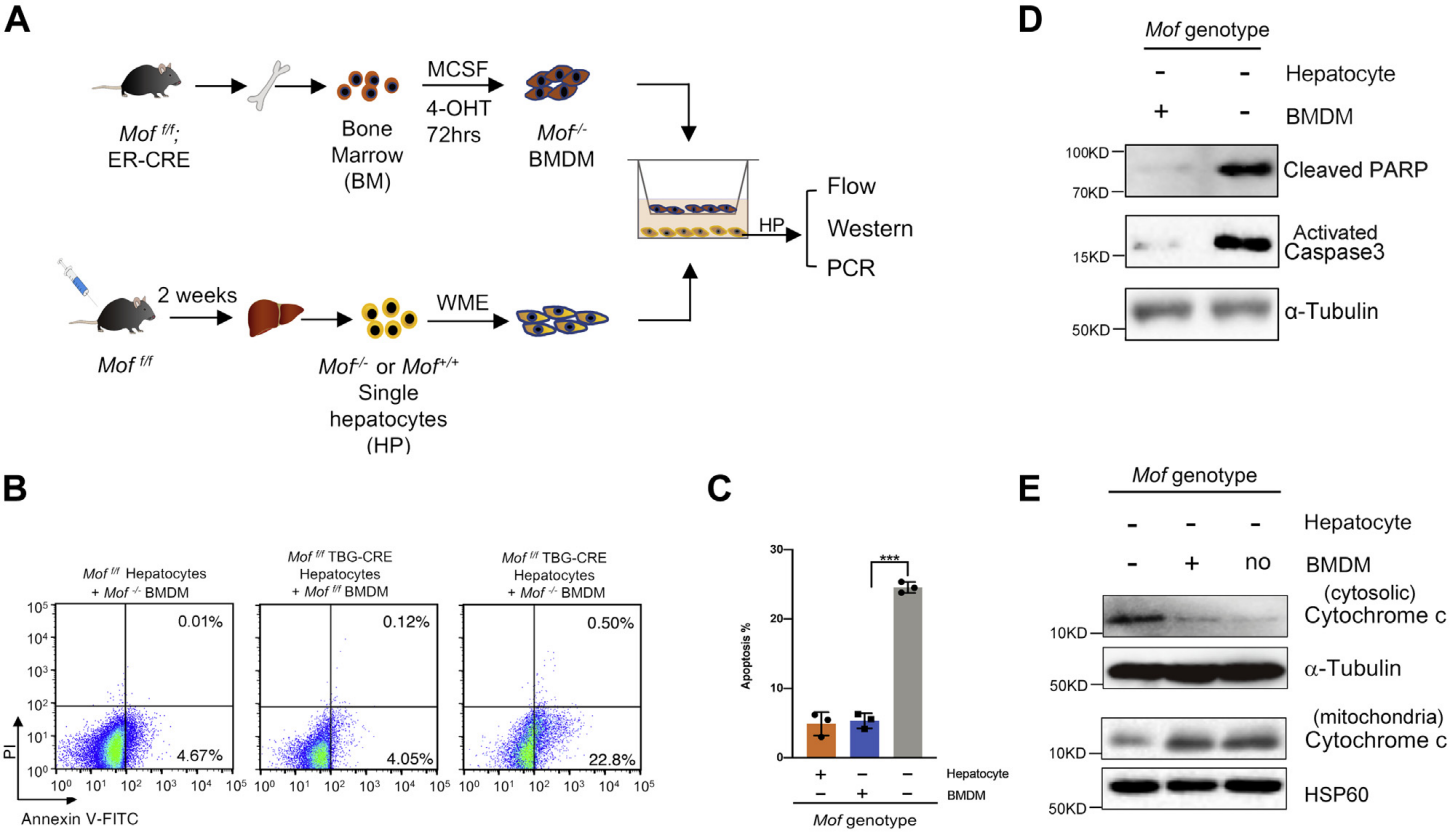
***Mof***^***−/−***^**BMDM coculture increased apoptotic response by *Mof***^***−/−***^**hepatocyte *in vitro*.***A*, the schematics for the coculture experiments. *B*, representative FACS analysis for Annexin V-FITC and PI stained hepatocytes after coculturing with *Mof*^*f/f*^ or *Mof*^*−/−*^ BMDM. Early apoptotic cells (AnnexinV^+^ and PI^−^) were shown in the lower right quadrant, and late apoptotic cells (AnnexinV^+^ and PI^+^) were shown in the upper right quadrant. *C*, scatter plot for quantification of the flow cytometry analyses for the coculture experiment as indicated at bottom. Mean and standard deviation (error bar) from three independent experiments were presented. (∗∗∗*p* < 0.001, student *t*-test). *D*, western blots for the cleaved PARP and activated caspase 3 proteins in *Mof*^*−/−*^ hepatocyte after coculture with *Mof*^*f/f*^ (left lane) or *Mof*^*−/−*^ (right lane) BMDM. Western blot for Tubulin was used as the loading control. *E*, western blot of cytosolic and mitochondria cytochrome c, indicated on right, in *Mof*^*−/−*^ hepatocytes after coculturing with *Mof*^*f/f*^, *Mof*^*−/−*^ or no BMDMs.

The discrepancy of *Mof* function in the Mx1-Cre and AAV-TBG-Cre mouse models suggests that *Mof* deletion-induced liver injury and steatohepatitis-like features probably require coordinated changes in both hepatocytes and *Kupffer* cells. To test this, we performed the *in vitro* coculture experiment using primary *Mof* ^*−/−*^ and *Mof* ^*f/f*^ hepatocytes with bone marrow–derived macrophages (BMDMs) isolated from ER-Cre; *Mof* ^*f/f*^ mice (Fig. 6*A*). The BMDM are commonly used to study the function of *Kupffer* cells *in vitro* (34). *Mof* deletion in the BMDM was induced by adding 100 nM tamoxifen (4-OHT) to the cell culture 72 h prior to the experiment (Fig. 6*A* and Fig. S5). Ethanol was used as the control for mock treatment. Simultaneous deletion of *Mof* in both hepatocytes and BMDM led to significant increase of apoptosis in hepatocytes (Fig. 6*B*, right), which is in contrast to that of *Mof* ^*f/f*^ hepatocytes cocultured with *Mof* ^*−/−*^ BMDM (Fig. 6*B*, left) or that of *Mof* ^*−/−*^ hepatocytes cocultured with mock-treated *Mof* ^*f/f*^ BMDM (Fig. 6*B*). Quantifications of the apoptosis analyses were shown in Figure 6*C*. Consistent with Annexin V/PI staining, the levels of cleaved PARP as well as active Caspase 3 proteins were significantly higher in *Mof* ^*−/−*^ hepatocytes after coculturing with *Mof* ^*−/−*^ BMDM than those after coculturing with mock-treated *Mof* ^*f/f*^ BMDM (Fig. 6*D*). Furthermore, cytosolic cytochrome c, a downstream effector of mitochondria apoptosis, was also significantly increased in *Mof* ^*−/−*^ hepatocytes after coculturing with *Mof* ^*−/−*^ BMDM, but not with mock-treated *Mof* ^*f/f*^ BMDM or no BMDM (Fig. 6*D*). Reciprocal changes of mitochondrial cytochrome c provided further confirmation (Fig. 6*E*). These results argue that liver injury observed in the Mx1-Cre; *Mof* ^*f/f*^ mice likely requires simultaneous loss of *Mof* in both cellular compartments *in vivo*.

### Reciprocal signaling between hepatocytes and BMDM is required for inflammation response

**Figure 7:**
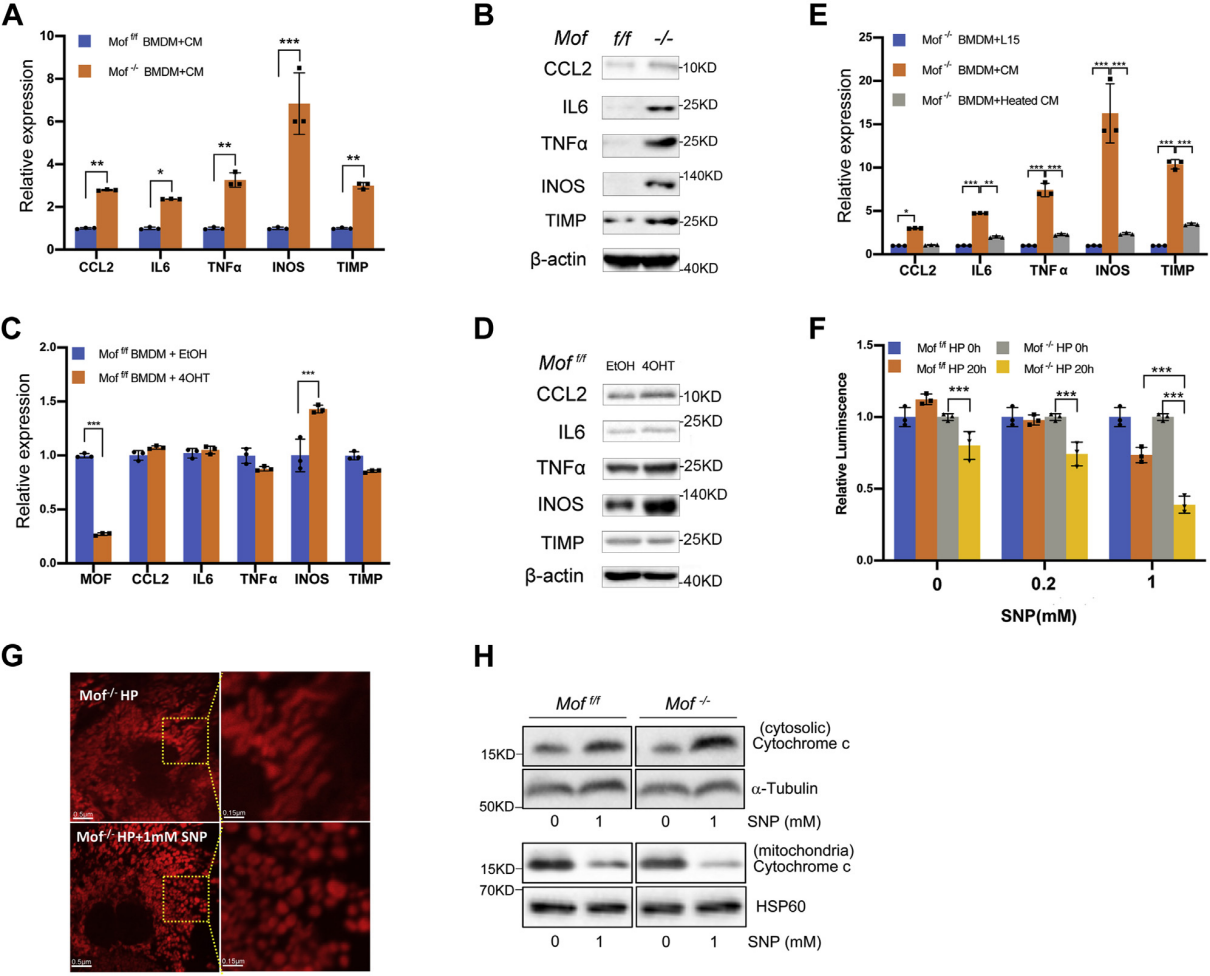
***Mof* regulates reciprocal signaling between BMDM and hepatocytes.***A*, relative gene expression in *Mof*^*f/f*^ and *Mof*^*−/−*^ BMDM after culturing with the conditioned medium from *Mof*^*−/−*^ hepatocytes. Y-axis is fold change after normalization against expression level of *Gapdh*, which is arbitrarily set as 1. Mean and standard deviation (error bar) from three independent experiments were shown. (∗*p* < 0.05; ∗∗*p* < 0.01, ∗∗∗*p* < 0.001, two-way ANOVA test). *B*, immunoblots for CCL2, IL6, TNFα, INOS, and TIMP in *Mof*^*f/f*^ (*left lane*) and *Mof*^*−/−*^ BMDMs (*right lane*) cultured in the *Mof*^*−/−*^ hepatocyte-conditioned medium. Immunoblot for β-actin was used as the loading control. *C*, relative gene expression in *Mof*^*f/f*^ BMDM after 100 mM 4OHT or EtOH treatment. Y-axis is fold change after normalization against *Gapdh level*, which was arbitrarily set as 1. Mean and standard deviation (error bar) from three independent experiments were shown. (∗∗∗*p* < 0.001, two-way ANOVA test). *D*, immunoblot for CCL2, IL6, TNFα, INOS, and TIMP in *Mof*^*f/f*^ BMDM after 100 mM 4OHT or EtOH treatment. Immunoblot for β-actin was included as the loading control. *E*, relative gene expression in *Mof*^*−/−*^ BMDM cultured in L15 media, the conditioned medium from *Mof*^*−/−*^ hepatocytes, or the conditioned medium after heating at 100 °C for 5 min. Y-axis is fold change after normalization against expression of *Gapdh*, which is arbitrarily set as 1. Mean and standard deviation (error bar) from three independent experiments were shown. (∗*p* < 0.05, ∗∗∗*p* < 0.001, two-way ANOVA test). *F*, cell-titer Glo assay for ATP production at 0 or 20 h after SNP treatment in primary *Mof*^*−/−*^ and *Mof*^*f/f*^ hepatocytes. Mean and standard deviation (error bar) from three independent experiments were presented. (∗∗∗*p* < 0.001, two-way ANOVA test). *G*, representative MitoTracker live staining of *Mof*^*−/−*^ hepatocytes with or without 1 mM SNP treatment, scale bar 0.5 μm. *Right panels* were enlargement of the square area from the left images (scale bar 0.15 μm). *H*, western blots for cytosolic or mitochondria cytochrome c in *Mof*^*−/−*^ hepatocytes with or without SNP treatment. Antibodies were indicated on the right.

To examine *Mof*-dependent signaling in the liver microenvironment that may contribute to liver injury, we first examined whether *Mof* ^*−/−*^ hepatocytes promote inflammation signaling to BMDM. To this end, we cultured the *Mof* ^*−/−*^ and *Mof* ^*f/f*^ BMDMs in the *Mof* ^*−/−*^ hepatocyte-conditioned medium. The *Mof* ^*−/−*^ BMDMs were activated by the *Mof* ^*−/−*^ hepatocyte-conditioned medium, expressing much higher level of chemokines CCL2, proinflammatory cytokine IL6, tumor necrosis factor (TNF)-α, profibrotic gene TIMP, and inducible nitric oxide synthase (iNOS) (Fig. 7*A*), consistent with *in vivo* RNA-seq analysis (Fig. 3*D*). Western blot analyses further confirmed changes of these genes at the protein levels (Fig. 7*B*). In contrast, *Mof* ^*−/−*^ BMDM or *Mof* ^*f/f*^ BMDMs cultured in regular medium had no or only modest change in CCL2, IL6, or TNFα at gene expression (Fig. 7*C*) and protein levels (Fig. 7*D*). Importantly, heat treatment of the *Mof* ^*−/−*^ hepatocyte-conditioned medium abolished its ability to activate *Mof* ^*−/−*^ BMDM (Fig. 7*E*). These results suggest that *Mof* null hepatocyte may provide cytokine signaling for macrophage activation.

We next examined the reciprocal signaling from macrophages to hepatocytes. *Mof* ^*−/−*^ BMDM expressed higher level of *iNOS* (Fig. 7, *C*–*D*), which increases release of nitric oxide (NO) to trigger apoptotic response (35, 36). We next asked whether *Mof* ^*−/−*^ hepatocytes are more sensitive to NO signaling and contribute to a feedback amplification of cell death signaling. Since primary BMDMs are difficult to transfect and have short viability *in vitro* for genetic studies, we directly treated *Mof* ^*f/f*^ and *Mof* ^*−/−*^ hepatocytes with sodium nitroprusside (SNP) as the NO donor. As shown in Figure 7*F*, although 1 mM SNP treatment decreased ATP production in both *Mof* ^*f/f*^ and *Mof*^*−/−*^ hepatocytes, *Mof*^*−/−*^ hepatocytes were much more sensitive to NO signaling than *Mof* ^*f/f*^ hepatocytes (Fig. 7*F*). Mitochondria in 1 mM SNP-treated *Mof* ^*−/−*^ hepatocytes were significantly smaller and more punctuated as compared with the mock-treated cells (Fig. 7*G*), indicative of onset of apoptosis (37, 38). Consistently, 1 mM SNP-treated *Mof* ^*−/−*^ cells released more cytochrome c from mitochondria into cytosol than that of the *Mof* ^*f/f*^ hepatocytes (Fig. 7*H*). These results suggest that *Mof* deletion probably altered the reciprocal signaling between hepatocytes and macrophages in the liver microenvironment, which leads to a feedforward amplification of the inflammation (BMDM) and cell death (hepatocyte) responses leading to liver injury and steatohepatitis-like features (see Discussion).

## Discussion

Here we find that *Mof* deletion by Mx1-Cre leads to severe liver injury with increasing lipid deposition and fibrosis. Notably, the liver injury and inflammation upon *Mof* deletion bear some similarity with human steatohepatitis at both phenotypical and molecular levels. Manifestation of liver injury in *Mof* null mice is heterogeneous. Majority of mice have accumulation of small lipid droplets in the liver, and a small subset of *Mof* null mice develop apparent fatty liver diseases. Significant heterogeneity of the nonacoholic fatty liver spectrum has also been widely reported in human patients (39, 40). About ∼20% patients have nonacoholic fatty liver or steatohepatitis without obesity or high-fat diet (39). It suggests that a multitude of factors (*e.g.*, diet, genetic and epigenetic factors) may contribute to the disease progression. Comparing with the natural progression of NAFLD/NASH in patients and the widely used high-fat diet mouse model, *Mof* null mice acutely develop steatohepatitis-like liver injuries. It is likely that downregulation of *MOF* is one of the key downstream events in progression of these deadly liver diseases. In this scenario, *MOF* downregulation may modulate the cellular epigenetic landscape to activate a feedback loop that leads to sustained inflammation and eventual liver injury. Indeed, we find significant downregulation of *MOF* in human terminal NASH patients as well as aberrant expression of a common subset of genes in both human NASH and the *Mof* null mouse models. To our knowledge, our study is the first one to demonstrate a causal role of a histone acetyltransferase in liver abnormalities.

A previous study reported that hematopoietic stem cells isolated from Mx1-Cre; *Mof* ^*f/f*^ mice were unable to sustain long-term hematopoiesis after transplantation into the recipient mice (41). In our study, we did not observe any hematopoietic defects in the primary mice for the duration of our study. In contrast to liver injury observed in the Mx1-Cre; *Mof* ^*f/f*^ mouse model, hepatocyte-specific deletion by AAV-TBG-Cre has no overt defects in adult livers. Similarly, myeloid-specific *Mof* deletion by Lyz2-Cre does not affect macrophage or hepatic functions in mice (42). Thus, severe liver injury in the Mx1-Cre is likely due to simultaneous deletion of *Mof* in both hepatocytes and *Kupffer* cells in liver. This is supported by the *in vitro* coculture experiments using hepatocytes and BMDMs. We have revealed a causal role of *Mof* in regulating the reciprocal signaling between BMDM and hepatocytes. *Mof* deletion in both BMDM and hepatocytes triggers an aberrant proinflammatory cascade that is not observed in its deletion in either cell compartment alone. Interestingly, *Mof* deletion in hepatocytes increases cytokine signaling for BMDM activation, as exemplified by increase of TNF-α and toll-like receptor (TLR) signaling (*e.g.*, TNFα, IL-6) (Fig. 7*A*). These signaling pathways have been reported as the major contributors to NASH progression in patients (43). The activated macrophages, in turn, release NO and other cytokines to induce apoptosis as well as proinflammatory responses in the liver as previously reported (44, 45). In the feedback loop, *Mof* deletion in hepatocytes also enhances sensitivity to NO-mediated death signaling (Fig. 7). Thus, our study highlights the necessity of coordinated epigenetic dysregulation in both hepatocytes and *Kupffer* cells during development of the steatohepatitis-like liver injury.

By examining genes that are dysregulated in both *Mof* null mice, we reveal that MOF is important for regulating multiple metabolic pathways, including lipid metabolism and oxidation–reduction process. The metabolic aberration likely leads to oxidative stress in the liver microenvironment, which further disrupts hepatic lipid and cholesterol synthesis, perpetuating a feedback loop that aggravates liver dysfunction (46). The lipotoxic and oxidative stress are able to trigger a cascade of proinflammatory events. Both metabolic dysregulation and inflammation are major contributors to NASH progression in patients (43). Notably, previous studies have shown that hepatocyte-specific deletion of histone deacetylase *Hdac3* and *Sirt1* leads to steatosis (47, 48). These histone deacetylases disrupt lipid and glucose homeostasis and reroute metabolic precursors toward lipid synthesis and lipid sequestration *in vivo* (47, 48). Our finding that *Mof* is also important to regulate lipid homeostasis, through modulating fatty acid oxidation (*e.g.*, Acss2, Acss3) and the cellular redox state, shows that the balance of histone acetylome is probably necessary to maintain metabolic homeostasis in the liver. Breaking the balance by depleting either histone acetyltransferase *Mof* or histone deacetylases will result in disruption of normal liver functions. Finally, it is worth noting that in addition to aggressive liver injury in the Mx1-Cre; *Mof* ^*f/f*^ mice, the gene pathway associated with cancer was also modestly enriched in *Mof* null liver and NASH patients (Fig. S4*A*). Given global downregulation of MOF and H4K16ac in HCC (23, 24), it would be of interest to examine whether MOF plays a role in the progression of NASH to HCC in future.

## Experimental procedures

### Mouse strains and genotyping strategies

Generation of *Mof* ^*f/f*^ and *Mof* ^*f/f*^; ER-Cre alleles were previously described (13). The Mx1-Cre; *Mof* ^*f/f*^ mice were generated by breeding the *Mof* ^*f/f*^ mice to B6.Cg-Tg(Mx1-cre)1Cgn/J mice (Jackson Laboratories, 003556). For *Mof* deletion in the Mx1-Cre model, polyI:C (Amersham) was intraperitoneally injected into mice at 2.5 μg/g concentration every other day for six consecutive doses. To generate hepatocyte-specific *Mof* deletion, 10^9^ pfu of either AAV-TBG-GFP (control) or AAV-TBG-Cre viruses (to ablate hepatic floxed genes) in 100 μl of sterile PBS were injected into tail vein of the 8-week-old *Mof* ^*f/f*^ mice. Gene deletion in the liver of the AAV-TBG-Cre-injected mice was achieved after 2 weeks. Genotyping strategies were previously described (15) and illustrated in Fig. S1*A* and Figure 5*B*. Briefly, mouse tails (5–10 mg) were boiled in 150 μl NaOH (50 mM) for 25 min, followed by addition of 15 μl Tris-HCl (1 M, pH 7.5). Genomic DNA was used as the template for PCR reaction using primers (F: TGCTCGTGGTAGTTGACAGC, R: TGGGCTCCAGGATAAACTTG). The reaction was carried out using cycling parameters: 94 °C for 2 min; 35 cycles of 94 °C for 30 s, 59 °C for 30 s and 72 °C for 30 s; followed by 72 °C for 2 min. Using this method, successful *Mof* deletion can be detected as an 850 kb band on the agarose gel (15). All animal experiments were performed in accordance with guidelines set by the Institutional Animal Care and Use Committee (IACUC) at the University of Michigan and University of Southern California. Animal experimentations were performed in strict accordance with the recommendations in the Guide for the Care and Use of Laboratory Animals of the National Institutes of Health. In all experiments, equal ratio of male and female mice was used, and *Mof* deletion does not confer sex bias in our study.

### Western blot analysis

Frozen liver tissues were ground in a mortar and pestle and lysed in the RIPA buffer (150 mM NaCl, 50 mmol/l Tris-HCl, pH 7.4, 1 mM EDTA, 1% Triton X-100 (Sigma-Aldrich), 1% sodium deoxycholic acid, 0.1% sodium dodecyl sulfate, 1 mM phenylmethylsulfonyl fluoride, and 1X cOmplete protease inhibitor cocktail (Thermo Fisher)). Primary hepatocytes were lysed in the RIPA buffer directly in the culture dish. Mitochondria were isolated from cells using the Mitochondria Isolation Kit (ThermoFisher, 89874). Protein concentrations were determined by the Bradford assay (BioRad) and analyzed on an Ultraspec 2100 Pro spectrophotometer (Amersham Biosciences) at 595 nm. Five micrograms of total protein was loaded on 4% to 20% Mini-Protean TGX Precast gels (BioRad) and transferred to membranes using the Trans-Blot Cell system (BioRad). The blots were probed with following primary antibodies: anti-α-Tubulin (1:5000; Sigma Aldrich, T9026), anti-β-Actin (1:5000; Sigma, A5441), anti-MOF (1:1000; Bethyl Laboratories, A300), anti-H4 (1:3000; Millipore, 07-329), anti- H4K16ac (1:5000; EMD Millipore, 07-329), anticytochrome C (1:1000; Abcam, ab13575), anticleaved PARP (1:1000; Cell Signaling Technology, 9548), anticleaved caspase-3 (1:1000; Cell Signaling Technology, 9661), anti-iNOS (1:1000; Cell Signaling Technology, 13120), anti-TNF-α (1:2000; Cell Signaling Technology, 3707), anti-CCL2 (1:1000; Cell Signaling Technology, 2029), anti-IL6 (1:1000; Cell Signaling Technology, 12912), anti-TIMP (1:500; sino-biological, 106164-T40), anti-HSP60 (1:3000; Cell Signaling Technology, 12165). The western blot was quantified by ImageJ.

### Hematoxylin and eosin, Masson’s trichrome, and Sirius Red staining

For hematoxylin and eosin (H&E) or Masson’s trichrome staining, livers were immersion-fixed with 10% buffered formalin and embedded in paraffin for sectioning. For the H&E staining, the deparaffinized slides were incubated by hematoxylin for 1 min followed by washing in flowing tap water for 5 min. The slides were then stained with eosin (Sigma HT110232) for 1 min, followed by wash with tap water and differentiation procedure. For the Masson’s trichrome staining, liver paraffin slides were subjected to Masson’s trichrome stain according to the manufacturer’s instructions (Trichrome Stain Masson Kit; Sigma-Aldrich; HT15). For the Sirius Red staining, the mouse liver paraffin slides were baked at 60 °C for 1 h and soaked in xylene and graded ethanol solutions (100%, 95%, 85%, 75%, 60%, 50% till 0%). Slides were then stained with 0.1% Sirius Red (Sigma, 365548) and 0.1% Fast Green (Sigma, F7252) (dissolved in saturated picric acid) overnight. The slides were washed with 10 mM hydrochloric acid for 2 min, rapidly dehydrated through graded alcohols starting at 70% and sealed with cover slips by Permount mounting medium.

### Immunofluorescence and immunohistochemistry

Livers were embedded with Tissue-Tek OCT compound (Sakura Finetek, Torrance, CA) and snap-freezed in liquid nitrogen. Cryostat sections were mounted on salinized slides and fixed with ice-cold acetone. Immunohistochemistry (IHC) for MOF was performed on cryostat sections using anti-MOF antibody from Abcam (ab2000660, 1:100 dilution). For immunofluorescence and Oil Red O staining, cryostat sections were mounted on salinized slides and fixed with ice-cold acetone. Fc receptors were blocked with 1% anti-mouse CD16/32 antibody (Biolegend, 10135) in 2% normal goat serum (NGS) for 30 min at room temperature. Slides were incubated with anti-Keratin 8 antibody (Lifespan Biosciences, LS-B7928), antiacetylated histone H4K16 (Abcam, ab109463), followed by Alexa-labeled secondary antibody (Invitrogen) at 1:200 dilution. Images were obtained using Olympus BX43 microscope and Cell Sens Software.

### Isolation and *in vitro* culture of primary hepatocytes

The liver was briefly perfused with the buffer containing Hank’s buffered saline (HBSS) without Ca^2+^/Mg^2+^, 720 μM EDTA, 0.075% Na_2_HCO_3_), followed by Collagenase II solution (HBSS with Ca^2+^/Mg^2+^, 0.075% Na_2_HCO_3_, 1 mM CaCl_2_, 1 mg/ml Collagenase Type II (Worthington, CLS-2)) at ∼1 ml/min for 5 min. After perfusion, livers were dissected and minced gently using forceps. Hepatocytes were washed with HBSS, filtered through 70 μm nylon cell strainer (Corning), and centrifuged at 50*g* for 3 min. Liver cell mixture was adjusted to 5 to 10 × 10^6^ cells/ml with complete L15 media (L15 pH 7.4, 0.72 mM HEPES, 60 mg/l glucose, and 5% FBS) and mixed with equal volume of Percoll solution (9:1 mixture of Percoll (GE, 17-0891-02) and 10XHBSS). The cell mixture was subject to centrifugation at 50*g* for 10 min at 4 °C. Hepatocytes were gently resuspended and washed with complete L15 media twice before plating on collagen-coated plates at the density of 1.2 × 10^6^/ml for culturing.

### Blood chemistry

For the Mx1-Cre mouse model, the blood was collected from tail vein or by cardiac puncture to the left ventricle of the euthanized mice at day 26 post PolyI:C treatment. For the AAV-TBG-Cre; *Mof* ^*f/f*^ mouse model, the blood was collected from tail vein at day 26 post AAV injection. In all cases, serum was isolated by centrifugation. Analysis of the liver panel (AST, AKT, LDH, Cholesterol, Creatinine, Bilirubin, Triglycerides, ALK phosphatase) was performed at the University of Michigan Unit for Laboratory Animal Medicine (ULAM) Laboratory.

### Flow cytometry

Peripheral blood was collected through tail vein and red blood cells were lysed using ACK lysis buffer (Lonza). Cells were washed with 1X phosphate buffered saline (PBS) and resuspended in buffer containing 1X PBS, 1% fetal bovine serum, and 2 mM EDTA. Terminally differentiated hematopoietic cells were stained with anti-CD3 (Biolegend, 1:200), anti-B220 (Biolegend, 1:200), anti-Gr1 (Biolegend, 1:200), or anti-CD11b (Biolegend, 1:200) antibodies for 1 h and analyzed on the BD LSRII flow cytometer. For apoptosis analysis, cells were collected, washed with PBS, and stained with Annexin-V FITC (BD Biosciences, 556420) and propidium iodide (PI) dye (ThermoFisher, 00-6990-50) for 15 min according to manufacturer's recommendations. After removal of excessive dye, cells were resuspended in 500 μl 1× PBS and analyzed by flow cytometry (BD FACSCanto II, BD Bioscience, USA). Unstained cells were used for the negative gating control. A total of 6000 events were recorded. The percentage of live, early apoptotic, late apoptotic, and necrotic cells were analyzed using the FlowJo software.

### Preparation of the hepatocyte-conditioned medium

For hepatocyte-conditioned medium, primary hepatocytes isolated from the AAV-TBG-Cre; *Mof* ^*f/f*^ mice were plated at the density of 1.2 × 10^6^/ml in L15 media. The medium was collected after 24 h and stored at −80 °C. For heat treatment, the conditioned medium was heated at 100 °C for 5 min. For the L929-conditioned medium, 2 × 10^5^ L929 cells were seeded with 150 ml DMEM supplemented with 100 units/ml penicillin and streptomycin, 1% L-glutamine, and 10% FBS (Life Technologies, 10082). The medium was collected twice with 7-day interval and filtered (0.22 μM) before storage at −80 °C.

### Isolation and activation of bone marrow–derived macrophages (BMDMs)

Primary bone marrow cells were isolated from the femur and tibia of the *Mof* ^*f/f*^; ER-Cre mice and cultured in DMEM supplemented with 20% L929 conditioned medium, 10% heat-inactivated FBS, 100 nM 4-OHT, 100 units/ml penicillin and streptomycin for 4 days to differentiate into bone marrow–derived macrophages (BMDMs). BMDMs were activated by culturing with 50% hepatocyte-conditioned medium for 18 h. For coculture experiment, 1 × 10^5^ BMDMs were seeded to an insert (Corning, CLS3428) with hepatocytes at bottom for 2 days before the experiment.

### Mitochondria staining

Cells were grown on 12-mm coverslips and stained with 250 nM Mitotracker Red CMXRos (M7512, Molecular Probes) for 30 min at 37 °C. The coverslips were washed twice with 1xPBS and three times with the growth medium. The cells were fixed with 4% formaldehyde for 15 min at room temperature (RT), permeabilized with 0.25% Triton X-100 for 30 min at 4 °C, and stained with DAPI for 15 min at room temperature before microscopy.

### RNA isolation, real-time quantitative PCR, and RNA-seq analysis

Total RNA was isolated from liver tissues, primary hepatocytes, or BMDMs using TRIzol (Invitrogen). A total 5 μg of RNA was reverse transcribed using SuperScript III Reverse Transcriptase (Invitrogen). Real-time quantitative PCR (RT-qPCR) was performed using Radiant Green 2x qPCR Mix Lo-ROX (Stellar Scientific) on a BioRad C1000 Touch ThermoCycler. Primers were listed in Table S4. All the statistical analysis for RT-qPCR was performed using GraphPad Prism 8 software. For RNA-seq analysis, triplicates of RNA were isolated, treated with DNase I. RNA integrity analysis was performed using an Agilent Bioanlyzer. Only RNA with RNA integrity numbers (RINs) of 8 or above was used to prepare libraries. The samples were sequenced on the Illumina HiSeq2000 platform. TopHat2 was used to map reads to mouse reference genome assembly mm9. Mapped reads were then analyzed by DESeq to identify differentially expressed genes. Gene expression was considered significantly different if 1) the adjusted *p* value was less than 0.05 and 2) log2 (fold change) was greater than 1 or less than −1. Volcano plots were generated using R software (http://www.r-project.org/). Clusters were identified using ClusterONE28 and analyzed for Gene Ontology (GO) terms using BinGO29. Unsupervised GO analysis was performed using all differentially expressed genes as input for DAVID (https://david.ncifcrf.gov) and visualized using GOPlot30 in R with false discovery rate (FDR) ≤ 0.05. KEGG pathway analysis was performed using up- or downregulated gene sets in DAVID. Only pathways with adjusted *p* value ≤0.05 were considered significant.

## Data availability

RNA-seq data for *Mof* ^*f/f*^ and *Mof* ^*−/−*^ liver tissues are deposited into NCBI’s Gene Expression Omnibus (GEO) with accession number GSE106369. RNA-seq data for primary human NASH patient samples are downloaded from data set GSE134422 (49) and GSE126848 (50).

## Conflict of interests

The authors declare no competing interests.

## Supporting information

Supplementary Figures and Tables

JBC_JBC/2020/016079_6.xlsx

JBC_JBC/2020/016079_7.xlsx

JBC_JBC/2020/016079_8.xlsx
