# Supplementary material for: Dysregulation of intercellular signaling by MOF deletion leads to liver injury
Source: J Biol Chem. 2021 Jan 7;296:100235. doi: 10.1074/jbc.RA120.016079 (PMC7948572; doi:10.1074/jbc.RA120.016079)
Supplement: Supplementary Figures and Tables [file mmc1.pdf]

SUPPLEMENTAL FIGURES

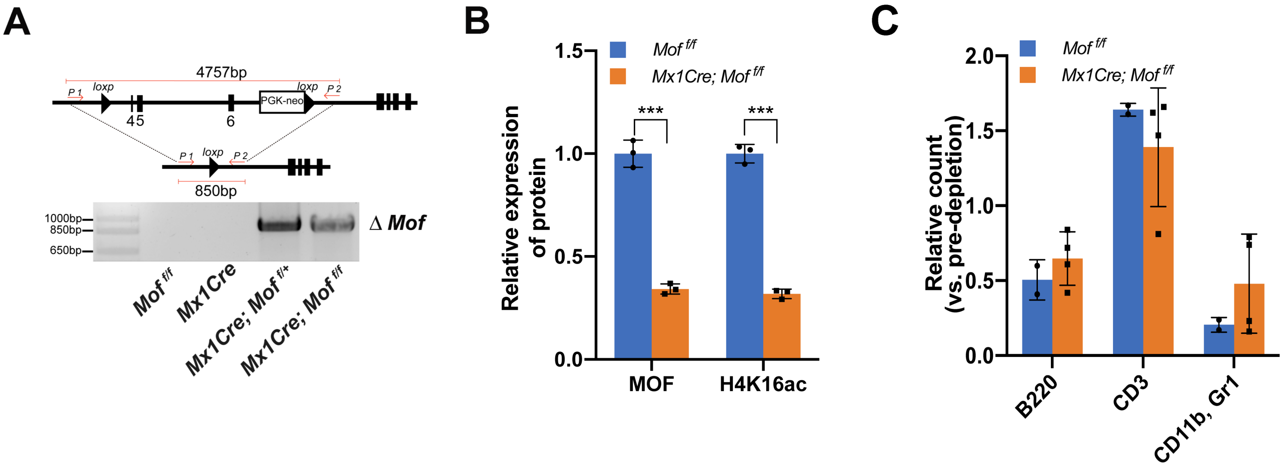

Supplemental Figure 1: Validation of *Mof* deletion in the *Mx1-Cre* mouse model and the effect on hematopoietic cells. (A) Top, genotyping strategy as previously described (1). *Mof* deletion is detected by PCR, which give rise to the 850bp product. Bottom, the agarose gel showing the DNA fragment indicative of *Mof* deletion in livers of the *Mof* $^{f/f}$, *Mx1Cre; Mof* $^{+/+}$, *Mx1Cre; Mof* $^{f/+}$ and *Mx1Cre; Mof* $^{f/f}$ mice after ploy I:C treatment. (B) Quantification of western blot shown Figure 1B by ImageJ. (***, $p<0.001$, two way ANOVA test). (C) Scatter plot for relative counts for B, T, and myeloid cells in peripheral blood from *Mof* $^{f/f}$ and *Mx1Cre; Mof* $^{f/f}$ mice at day 60 post poly I:C treatment. The fold change was presented after normalization against cell counts in individual animals prior to poly I:C treatment, which were arbitrarily set as 1.

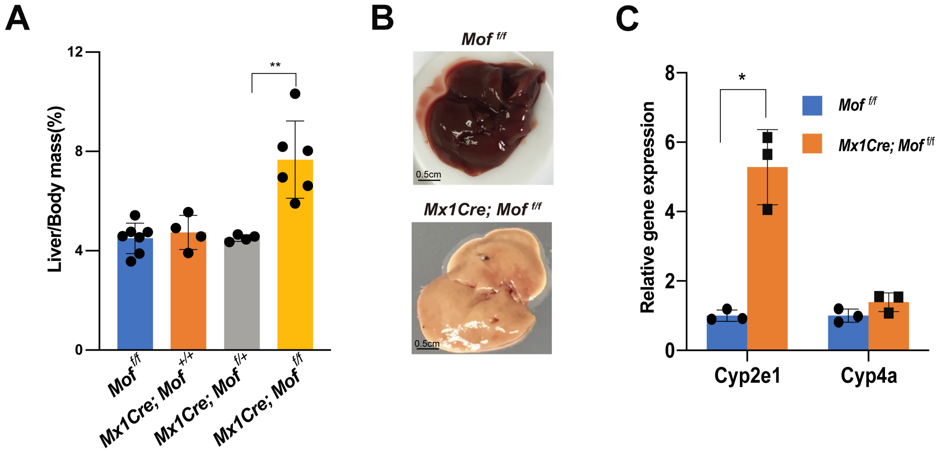

Supplemental Figure 2: *Mof* deletion by *Mx1Cre* leads to acute liver injury. (A) Comparison of liver to body mass ratios for *Mof* $^{f/f}$, *Mx1Cre; Mof* $^{+/+}$, *Mx1Cre; Mof* $^{f/+}$ and *Mx1Cre*; *Mof* $^{f/f}$ mice post poly I:C treatment. Y-axis, % of liver relative to body weight. (**, p<0.002, unpaired t test). (B) Images of whole livers isolated from polyI:C treated *Mof* $^{f/f}$ and *Mx1Cre; Mof* $^{f/f}$ mice at day 30. Livers from two *Mx1Cre; Mof* $^{f/f}$ mice showed significant whitening due to lipid deposition, scale bar 0.5 cm. (C) Relative gene expression of Cyp2e1 and Cyp4a, two indicators of lipotoxicity, in poly I:C treated *Mof* $^{f/f}$ and *Mx1Cre*; *Mof* $^{f/f}$ mice. Average fold change from three independent experiments in *Mx1Cre*; *Mof* $^{f/f}$ liver was presented after normalization against respective expression in the poly I:C treated *Mof* $^{f/f}$ liver, which was arbitrarily set as 1. Error bars represent standard deviation from three independent experiments. (*, p<0.033, two-way ANOVA).

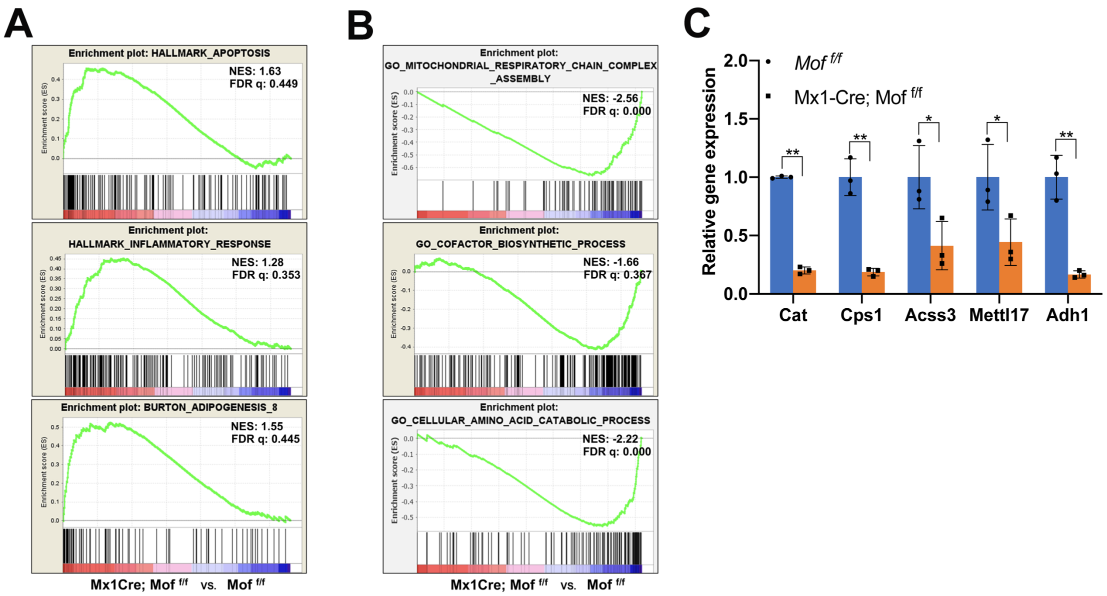

Supplemental Figure 3: Gene expression analysis for livers isolated from poly I:C treated *Mof* $^{f/f}$ and Mx1-Cre; *Mof* $^{f/f}$ cells. (A) GESA analysis for genes up regulated after Mof deletion in the *Mx1-Cre; Mof* $^{f/f}$ liver. Pathways such as apoptosis, inflammatory and fibroblast were significantly enriched upon *Mof* deletion. (B) GESA analysis for genes down regulated after Mof deletion in the *Mx1-Cre; Mof* $^{f/f}$ liver. Pathways, such as respiratory chain, fatty acid and amino acid, were significantly enriched upon *Mof* deletion. (C) Validation of the RNA-seq results. Selected genes in the fatty acid oxidation pathways were tested by real-time PCR. The fold change in gene expression in the *Mx1-Cre*; *Mof* $^{f/f}$ liver was presented after normalization against their respective expression in poly I:C treated *Mof* $^{f/f}$ liver, which was arbitrarily set as 1. Average fold change from three independent experiments were presented. Error bars represent standard deviation. (*, $p<0.05$, **, $p<0.01$, two-way ANOVA test).

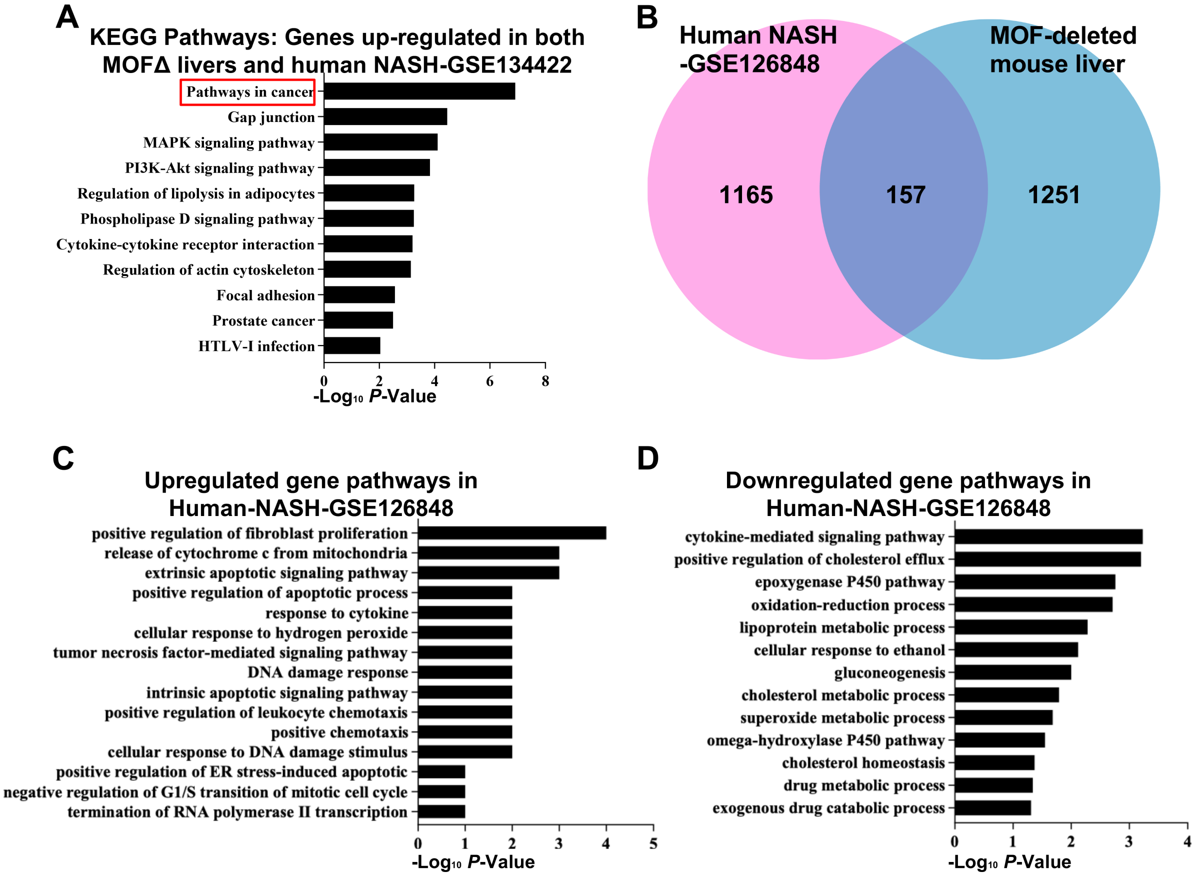

Supplemental Figure 4: Correlation of differential gene expression in human NASH patients and *Mof* KO mice. (A) KEGG pathway analysis for up-regulated genes in both *Mof* KO mouse livers and human NASH patients (GSE134422). Pathways in cancer is one of the top enriched KEGG pathways. (B) Venn diagram for differentially expressed genes in livers of *Mof* null mice (vs. *Mof* WT) and human NASH patients (vs. normal human subjects) (GSE126848). (C, D) Up (C) or down (D) regulated biological processes for differentially expressed genes in human NASH patients vs. normal human subjects.

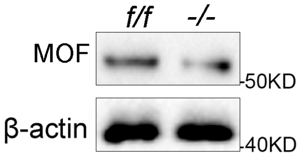

Supplemental Figure 5: Immunoblots for MOF after 4-OHT-mediated deletion in BMDM derived from ER-Cre; *Mof* $^{f/f}$ mice. Antibodies were labeled on right.

Supplemental Tables 1 to 4

Supplemental Table 1: RNA-seq analysis for mouse livers before and after *Mof* deletion.

Supplemental Table 2: Go-term biological processes analysis for human NASH dataset GSE134422 and its overlap with *Mof* null mouse liver.

Supplemental Table 3: Go-term biological processes analysis for human NASH dataset GSE126848.

Supplemental Table 4: Primer sequences used in this study.

**Gene (protein) for qPCR**	**Forward primer (5' -> 3')**	**Reverse primer (5' -> 3')**
**Mof**	GCGATCACCAAAGTGAAGTATGTA	GGTAGCTCTTCTCGAACTTCATGT
**CCL2**	ACTGAAGCCAGCTCTCTCTTCCTC	TTCCTTCTTGGGGTCAGCACAGAC
**IL6**	AGCCAGAGTCCTTCAGA	GGTCCTTAGCCACTCCT
**TNFα**	CATCTTCTCAAAATTCGAGTGACAA	TGGGAGTAGACAAGGTACAACCC
**iNOS**	CAGGGCCACCTCTACATTTG	TGCCCCATAGGAAAAGACTG
**TIMP**	GCTAAATTCATGGGTTCCCCAG	GAGAAAGCTCTTTGCTGAGCAG
**CYP4A**	TTTAGCCCTACAAGGTACTTGGA	GCAGCCACTGCCTTCGTAA
**CYP2E1**	CGTTGCCTTGCTTGTCTGGA	AAGAAAGGAATTGGGAAAGGTCC
**GAPDH**	CGACCACTTTGTCAAGCTCA	AGGGGTCTACATGGCAACTG
**Gene (protein) for genotyping**	**Forward primer (5' -> 3')**	**Reverse primer (5' -> 3')**
**Mof**	TGCTCGTGGTAGTTGACAGC	TGGGCTCCAGGATAAACTTG

Reference:

1. Li, X., Corsa, C. A. S., Pan, P. W., Wu, L., Ferguson, D., Yu, X., Min, J., and Dou, Y. (2010) MOF and H4 K16 acetylation play important roles in DNA damage repair by modulating recruitment of DNA damage repair protein Mdc1. Molecular and cellular biology 30, 5335-5347
